# Spontaneous splenic rupture in patient with metastatic melanoma treated with vemurafenib

**DOI:** 10.1186/1477-7819-10-155

**Published:** 2012-07-30

**Authors:** Elisa Castellani, Piero Covarelli, Carlo Boselli, Roberto Cirocchi, Antonio Rulli, Francesco Barberini, Daniela Caracappa, Carla Cini, Jacopo Desiderio, Gloria Burini, Giuseppe Noya

**Affiliations:** 1 Department of General and Oncologic Surgery, University of Perugia, Perugia, Italy; 2 Department of General Surgery, University of Perugia, St. Maria Hospital, Via Tristano di Joannuccio, Terni, 05100, Italy

**Keywords:** Spontaneous splenic rupture, Melanoma, BRAF, Vemurafenib

## Abstract

**Background:**

BRAF inhibitors such as vemurafenib are a new family of biological drugs, recently available to treat metastatic malignant melanoma.

**Methods:**

We present the case of a 38-year-old man affected by metastatic melanoma who had been under treatment with vemurafenib for a few days. The patient suffered from sudden onset of abdominal pain due to intra-abdominal hemorrhage with profuse hemoperitoneum. An emergency abdominal sonography confirmed the clinical suspicion of a splenic rupture.

**Results:**

The intraoperative finding was hemoperitoneum due to splenic two-step rupture and splenectomy was therefore performed. Histopathology confirmed splenic hematoma and capsule laceration, in the absence of metastasis.

**Conclusions:**

This report describes the occurrence of a previously unreported adverse event in a patient with stage IV melanoma receiving vemurafenib.

## Background

Atraumatic splenic rupture (ASR) is a rare pathology that can be idiopathic (7%) or pathological (93%) in nature [1]. While in the first group there are no abnormal histopathological findings, in the second one several etiological factors can be identified, including neoplastic (30%), infectious (27%), inflammatory non-infectious (20%), drug- or treatment-related (9%), and mechanical disorders (6%) [1].

ASR is an often unrecognized and potentially fatal cause of acute abdomen development; it should be routinely considered in the differential diagnosis of such pathology and when present promptly managed, at best with laparotomic approach [2]. The commonest causes of this rare pathology are: malignant hematological disorders (16%) such as acute leukemia and non-Hodgkin’s lymphoma; viral infections (15%) as infectious mononucleosis and cytomegalovirus infection; and local inflammatory and neoplastic disorders (11%) such as acute and chronic pancreatitis. Splenomegaly is a common feature, affecting 55% of patients. There is a male gender prevalence of 2:1, with a mean age of 45 years, (median 45, range 18–86 years). The overall ASR-related mortality rate is 12% [1].

Although, nowadays, the laparoscopic approach is the routine procedure for various diseases requiring elective splenectomy, in emergency cases laparotomy still retains its effectiveness, first and foremost with regard to patients with unstable hemodynamic conditions.

## Case presentation

In September 2011, a 38-year-old man was admitted in urgency regimen to our department because of a moderate left flank pain starting 6 h before hospital admission. The patient also presented vomiting and diffuse abdominal tenderness. Minor discomfort had been present in the left flank for a few days. The patient had a 3-year history of melanoma, starting in November 2008 with the removal of a forehead superficial spreading melanoma, 7 mm in diameter, Breslow thickness being 0.44 mm, with discrete lymphocytic infiltrate mainly in the surrounding tissue, without ulceration or regression. Only radicalization of local excision and follow-up were required according to the initial staging; sentinel node biopsy was therefore not performed.

In June 2011, 31 months after primary tumor excision, and 3 months before the episode of acute abdominal pain, the patient underwent an emergency orchiectomy because of the onset of acute scrotal swelling, with a pathologic diagnosis of metastatic melanoma to the testis (with an immunohistochemical profile positive for HMB-45, S-100, MART-1, and negative for pancytokeratin and inhibin). At that time, PET and CT scans showed several possible metastases, in soft tissues, gastro-intestinal tract, lungs, and brain; biopsies of masseter muscle, stomach, and duodenum confirmed the diagnosis.

The patient, after testing positive on screening for MO 255515 protein, was recruited to an experimental protocol using BRAF inhibitors and thus received a first cycle of therapy with vemurafenib at an oral dose of 960 mg twice daily. He did not undergo combined therapy with cytotoxic drugs, but received vemurafenib alone. Such experimental protocol was part of a compassionate care strategy and foresaw specific exclusion criteria, such as the presence of second malignancies, renal failure and diabetes mellitus, co-morbidities that our patient did not have. The acute abdominal event occurred 12 days after starting treatment, when the patient had assumed a total cumulative dose of about 23 g.

**Figure 1:**
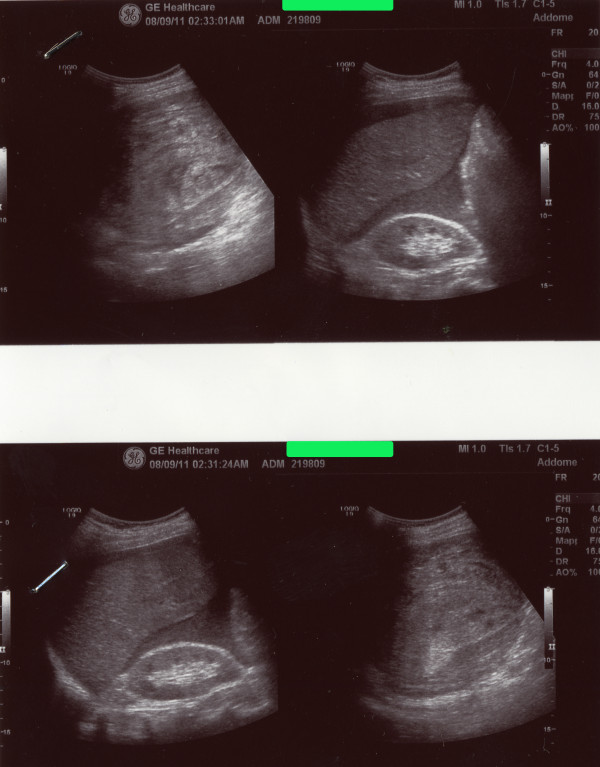
Abdominal ultrasonography showing the spleen rupture with hemoperitoneum.

**Figure 2:**
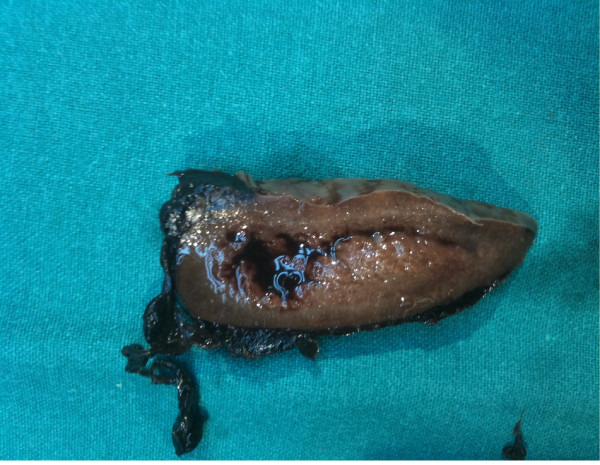
Section of spleen in which can be seen laceration of the capsule due to rupture in two times.

On admission the patient was pale and suffering, and his vital parameters were as follows: oxygen saturation 100%; heart rate 90 bpm, temperature 36.5°C, blood pressure 90/45 mmHg. His medical history was otherwise negative, and no trauma or accident had been previously reported. An abdominal examination showed diffuse pain and peritoneal fluid was noticed at percussion; a nasogastric tube was placed without detecting gastric retention and a urinary catheterization showed oliguria. A complete blood count showed moderate leukocytosis (13.67 WBC x10^3^) and anemia (RBC 3.42 x10^6^, Hb 10.4 g/dL, Ht 30.8%). An immediate abdominal sonography confirmed the clinical suspicion of spontaneous rupture of the spleen with massive hemoperitoneum (Figure 1). The patient immediately underwent an emergency laparotomy. Intraoperative findings have been massive hemoperitoneum (approximately 3 L of blood and coagula) due to a two-step rupture of the spleen, with subsequent subcapsular hematoma and complete decortication, that required a splenectomy; splenic size was within the normal range (Figure 2). Some suspected metastatic nodes were removed from the great omentum; histopathology confirmed the metastatic nature of the omental nodes while the spleen was decorticated in the absence of metastases.

The postoperative course was complicated by the onset, on the fourth day, of partial thrombosis of the great saphenous vein; the diagnosis was confirmed by sonography and a therapy of 40 mg low molecular weight heparin (LMWH) (Clexane®, Aventis Pharma, Bad Soden, Germany; 0.4 mL prefilled syringes) subcutaneously administered twice daily was undertaken. The abdominal drainage was removed 5 days after the operation, and the patient was discharged on the following day.

## Discussions

Melanoma is the deadliest form of skin cancer; its incidence has been increasing continuously and over 40,000 people die of this disease each year worldwide [3]. The estimated number of new cases of melanoma in 2010 in the United States was 68,130, of which 57% affected males and 43% females. In the same period, the estimated deaths caused by this pathology were 8,700 (65% males and 35% females) [4].

The disease presents genetically and clinically distinct subgroups that could indicate the need of patient-specific management strategies [5]. For example, head and neck mucosal malignant melanomas behave much more aggressively than limb or trunk tumors and their prognostic markers have not yet been fully elucidated [6]. The majority of metastases appears within 3 years from the diagnosis of primary melanoma [7]. Primary melanoma metastasizes most frequently to lymph nodes and lungs (about 70%). Less common sites of metastasis are liver (58%), brain (54%), bones (48%), adrenal glands (46%), and gastrointestinal tract (43%). Among this group, small intestine and spleen are affected in 36.5% and 30.6% of cases, respectively [7]. The 1-year survival rate for patients with systemic metastasis from cutaneous melanoma ranged from 40% to 60% in the American Joint Committee on Cancer (AJCC) Melanoma Staging Database [8]. Our patient had a stage IV melanoma, and, therefore, an extremely poor prognosis with median survival ranging from 6 to 18 months after diagnosis [9] and a 5-year survival rate of less than 5% [10-12].

Melanoma treatment has experienced a rapid change in recent years through recent discoveries of target therapies and immunotherapeutic antibodies. With the continuous progress of research, prolonged therapeutic success may be achieved through the tailored use of molecular markers and immunotherapies in sequential or combinatorial methods [13,14]. Advanced melanomas often have multiple genetic defects affecting several biochemical pathways. Somatic point mutations in RAF occur in approximately 8% of human tumors, most frequently in melanoma, colorectal, and thyroid cancers [4,14,15]. The targeting of this single oncogenic alteration with specific inhibitory nucleic acids or chemical RAF inhibitors in melanoma cell lines showed growth arrest and induction of apoptosis [16,17].

Although melanoma metastases have been found to contain thousands of mutations, the V600E BRAF mutation is clearly a driver of the neoplastic phenotype and is present in approximately 50% of melanomas [5,18]. The discovery of activating mutations (V600E) in the BRAF kinase encouraged the development of compounds to inhibit aberrant BRAF activity [5]. Vemurafenib (also known as PLX4032, RG7204, or RO5185426) marketed as Zelboraf is a BRAF enzyme inhibitor developed by Plexxikon (Berkeley, CA, USA, now part of the Daiichi Sankyo Group) and Roche [15]. PLX4032 was recently observed to increase median overall survival in metastatic melanoma. However, resistance through up-regulation of receptors or by activating mutations in oncogenic signaling and alternative enzymes is proving to be an emerging problem [3]. The comparison between vemurafenib and conventional treatment with dacarbazine led to encouraging results regarding 6-month survival, which was 84% in the vemurafenib group and 64% in the dacarbazine group. The risk of either death or disease progression was also reduced in the vemurafenib group [9]. In summary, these studies reveal that, for the first time, several immunotherapeutic and targeted agents are yielding some clinical responses and improvements in overall survival in patients with unresectable stage III and IV melanoma [3]. In patients with metastatic melanoma featuring BRAF V600E mutation, phase 1 and 2 clinical trials of the BRAF kinase inhibitor vemurafenib (PLX4032) have shown response rates of more than 50% [9].

Selective BRAF inhibitors show good tolerability with infrequent severe toxicities. Among the common adverse events associated with vemurafenib are skin changes 50% to 70% (rash, alopecia, keratoacanthoma or squamous-cell carcinoma, photosensitivity), fatigue, and arthralgia 30% to 50%, diarrhea 10% to 30%, and nausea 10% to 20% [16]. About 15% to 30% of patients treated with type I BRAF inhibitors, such as vemurafenib, develop squamous-cell carcinomas and keratoacanthomas. These non-melanoma skin cancers are not deadly, but they can become dangerous, when they rarely become metastatic [19]. Squamous cell tumors from patients treated with BRAF inhibitors have a distinct mutational profile. A higher frequency of activating RAS mutations was found in tumors from patients treated with vemurafenib (about 60%) *vs.* sporadic cases (range between 3% and 30%) [20]. Vemurafenib alone does not increase the number of tumors; it just decreases their latency by promoting pre-existent mutations. This is evidenced by the early appearance of lesions (within the first few weeks) after assumption of vemurafenib, and only in a subset of patients [21]. Therefore, testing the RAS status should be useful in patients who undergo treatment with BRAF inhibitors. Assuming that concomitant administration of MEK inhibitors can stop vemurafenib-induced acceleration of tumor growth in patients with RAS mutation, it may be possible to develop a new generation of BRAF inhibitors [22-24].

Another emerging problem is the resistance to BRAF inhibitors that develops within months [25].

Recent studies suggest that it could depend on tyrosine-kinase receptors (like PDGFR and IGFR-1) [26,27]. Inhibitors of c-Kit and mitogen-activated protein kinase (MEK) have also been found to act against melanomas, and MEK inhibitors are now being examined as a strategy to overcome BRAF inhibitor resistance [27].

ASR is uncommon but fatal if untreated. Its diagnosis should not be missed or delayed because of low clinical suspicion; in contrast it should be routinely considered in the differential diagnosis of acute surgical abdomen and of a wide range of medical conditions [28]. Various hypotheses could be advanced concerning the possible correlations between underlying disease, drug therapy, and acute complications. The possibility that a link between cancer and spontaneous splenic rupture exists in the absence of splenic metastasis or chemotherapy has been suggested for a long time, and the reasons are to be found in a hypercoagulable state secondary to the underlying malignancy [29-32]. Furthermore, the possibility of splenic rupture without a previous trauma in patients under treatment for abdominal manifestations of metastatic cancer has already been described, but metastases were assumed to be the cause [33,34]. Another possible cause might be found in alterations of angiogenesis pathways; BRAFV600E-dependent VEGF production has been suggested as angiogenetic promoter mechanism [35]. Oncogenic BRAF V600E mutation seems to enhance the expression of several proangiogenic and proinflammatory molecules, including VEGF-A [36]. BRAF binds to and is downstream from the main effectors of KRAS, whose activating mutations are believed to support the chaotic tumor vascularity, by up-regulating the transcription of several angiogenic inducers, including VEGF-A [37]. This might have caused splenic parenchyma fragility, resulting in a greater tendency to a spontaneous or minor trauma-related rupture; in fact, whether and to what measure BRAF regulates and alters angiogenesis is still unclear.

## Conclusions

As shown by clinical data, we can assume a relationship, which is currently not verifiable, between the intake of BRAF inhibitors and spontaneous rupture of the spleen; also superficial venous thrombosis in the postoperative course has been reported. With this report we intend to comment on an unusual event, namely the spontaneous rupture of the spleen occurred in a patient with stage IV melanoma under treatment with vemurafenib, in the absence of neoplastic involvement of the spleen, splenomegaly, or major alterations of coagulation.

## Consent

Written informed consent was obtained from the patient for publication of this manuscript and any accompanying images. A copy of the written consent is available for review by the Editor-in-Chief of this journal.

## Abbreviations

AJCC: American Joint Committee on Cancer; ASR: Atraumatic Splenic Rupture; BRAF: V-raf murine sarcoma viral oncogene homolog B1; CT: Computer Tomography; Hb: Hemoglobin; HMB-45: Human Melanoma Black; Ht: Hematocrit; IGFR-1: Insulin-like Growth Factor 1 (IGF-1) Receptor; IU: International Units; LMWH: Low Molecular Weight Heparin; MART-1: Melanoma Antigen Recognized by T-cells; MEK: Mitogen-Activated protein Kinase; PDGFR: Platelet-Derived Growth Factor (PDGF) Receptors; PET: Positron Emission Tomography; RBC: Red Blood Cells; S-100: 100% Soluble protein (in ammonium); WBC: White Blood Cells.

## Competing interests

The authors state that none of the authors involved in the manuscript preparation has any conflicts of interest regarding the manuscript itself, neither financial nor moral conflicts. Furthermore, none of the authors received support in the form of grants, equipment, and/or pharmaceutical items.

## Authors’ contributions

All authors contributed equally to this work, read, and approved the final manuscript.

## References

[B1] Renzulli P Hostettler A Schoepfer AM Gloor B Candinas D Systematic review of atraumatic splenic rupture Br J Surg 2009 96 1114 1121 10.1002/bjs.6737 19787754

[B2] Rhee SJ Sheena Y Imber C Spontaneous rupture of the spleen: a rare but important differential of an acute abdomen Am J Emerg Med 2008 26 733 18606341 10.1016/j.ajem.2007.11.003

[B3] Natarajan N Telang S Miller D Chesney J Novel immunotherapeutic agents and small molecule antagonists of signalling kinases for the treatment of metastatic melanoma Drugs 2011 71 1233 1250 10.2165/11591380-000000000-00000 21770473

[B4] Jemal A Siegel R Xu J Ward E Cancer statistics, 2010 CA Cancer J Clin 2010 60 277 300 10.3322/caac.20073 20610543

[B5] Vultur A Villanueva J Herlyn M Targeting BRAF in advanced melanoma: a first step toward manageable disease Clin Cancer Res 2011 17 1658 1663 10.1158/1078-0432.CCR-10-0174 21447722 PMC3079374

[B6] Kerr EH Hameed O Lewis JS Jr Bartolucci AA Wang D Said-Al-Naief N Head and neck mucosal malignant melanoma: clinicopathologic correlation with contemporary review of prognostic indicators Int J Surg Pathol 2012 20 1 37 46 10.1177/1066896911417970 21862490

[B7] Di Libero L Sciascia V Esposito D Varriale R Tartaglia E Santini L Surgical treatment of metastases from cutaneous melanoma to the small intestine and the spleen Case reports and review of the literature. Ann Ital Chir 2011 82 233 238 21780568

[B8] Balch CM Gerschenwald JE Soong S Thompson JF Atkins MB Byrd DR Buzaid AC Cochran AJ Coit DG Ding S Eggermont AM Flaherty KT Gimotty PA Kirkwood JM McMasters KM Mihm MC Jr Morton DL Ross MI Sober AJ Sondak VK Final version of 2009 AJCC melanoma staging and classification J Clin Oncol 2009 27 6199 6206 10.1200/JCO.2009.23.4799 19917835 PMC2793035

[B9] Chapman PB Hauschild A Robert C Haanen JB Ascierto P Larkin J Dummer R Garbe C Testori A Maio M Hogg D Lorigan P Lebbe C Jouary T Schadendorf D Ribas A O’Day SJ Sosman JA Kirkwood JM Eggermont AM Dreno B Nolop K Li J Nelson B Hou J Lee RJ Flaherty KT McArthur GA BRIM-3 Study Group: Improved survival with vemurafenib in melanoma with BRAF V600E mutation N Engl J Med 2011 364 2507 2516 10.1056/NEJMoa1103782 21639808 PMC3549296

[B10] Pollack LA Li J Berkowitz Z Weir HK Wu XC Ajani UA Ekwueme DU Li C Pollack BP Melanoma survival in the United States, 1992 to 2005 J Am Acad Dermatol 2011 Suppl 1 s78 86 22018071 10.1016/j.jaad.2011.05.030 PMC4890628

[B11] Hafner C Therapie des metastasierten Melanoms mit BRAF-Inhibitoren Hautarzt 2011 62 696 698 10.1007/s00105-011-2232-8 21863388

[B12] Rondelli F Vedovati MC Becattini C Tomassini GM Messina S Noya G Simonetti S Covarelli P Prognostic role of sentinel node biopsy in patients with thick melanoma: a meta-analysis J Eur Acad Dermatol Venereol 2012 26 5 560 565 10.1111/j.1468-3083.2011.04109.x 21561487

[B13] Hamid O Boasberg PD Rosenthal K O’Day SJ Systemic treatment of metastatic melanoma: New approaches J Surg Oncology 2011 104 425 429 10.1002/jso.22034 21858838

[B14] Pratilas CA Solit DB Targeting the mitogen-activated protein kinase pathway: physiological feedback and drug response Clin Cancer Res 2010 16 3329 3334 10.1158/1078-0432.CCR-09-3064 20472680 PMC2912210

[B15] Bollag G Hirth P Tsai J Zhang J Ibrahim PN Cho H Spevak W Zhang Y Habets G Burton EA Wong B Tsang G West BL Powell B Shellooe R Marimuthu A Nguyen H Zhan KY Artis DR Schlessinger J Su F Higgins B Iyer R D’Andrea K Koehler A Stumm M Lin PS Lee RJ Grippo J Puzanov I Clinical efficacy of a RAF inhibitor needs broad target blockade in BRAF- mutant melanoma Nature 2010 467 596 599 10.1038/nature09454 20823850 PMC2948082

[B16] Arkenau HT Kefford R Long GV Targeting BRAF for patients with melanoma Br J Cancer 2011 104 392 398 10.1038/sj.bjc.6606030 21139585 PMC3049553

[B17] Calipel A Lefevre G Pouponnot C Mouriaux F Eychene A Mascarelli F Mutation of B-Raf in Human choroidal melanoma cells mediates cell proliferation and transformation through the MEK/ERK pathway J Biol Chem 2003 278 42409 42418 10.1074/jbc.M308709200 12917419

[B18] Davies H Bignell GR Cox C Stephens P Edkins S Clegg S Teague J Woffendin H Garnett MJ Bottomley W Davis N Dicks E Ewing R Floyd J Gray K Hall S Hawes R Hughes J Kosmidou V Menzies A Mould C Parker A Stevens C Watt S Hooper S Wilson R Jayatilake H Gusterson BA Cooper C Shipley J Mutations of the BRAF gene in human cancer Nature 2002 417 949 954 10.1038/nature00766 12068308

[B19] Flaherty KT Puzanov I Kim KB Ribas A McArthur GA Sosman JA O’Dwyer PJ Lee RJ Grippo JF Nolop K Chapman PB Inhibition of mutated, activated BRAF in metastatic melanoma N Engl J Med 2010 363 809 819 10.1056/NEJMoa1002011 20818844 PMC3724529

[B20] Oberholzer PA Kee D Dziunycz P Sucker A Kamsukom N Jones R Roden C Chalk CJ Ardlie K Palescandolo E Piris A Macconaill LE Robert C Hofbauer GF McArthur GA Schadendorf D Garraway LA RAS mutations are associated with the development of cutaneous squamous cell tumors in patients treated with RAF inhibitors J Clin Oncol 2012 30 316 321 10.1200/JCO.2011.36.7680 22067401 PMC3269955

[B21] Su F Viros A Milagre C Trunzer K Bollag G Spleiss O Reis-Filho JS Kong X Koya RC Flaherty KT Chapman PB Kim MJ Hayward R Martin M Yang H Wang Q Hilton H Hang JS Noe J Lambros M Geyer F Dhomen N Niculescu-Duvaz I Zambon A Niculescu-Duvaz D Preece N Robert L Otte NJ Mok S Kee D RAS mutations in cutaneous squamous-cell carcinomas in patients treated with BRAF inhibitors N Engl J Med 2012 366 207 15 10.1056/NEJMoa1105358 22256804 PMC3724537

[B22] Poulikakos PI Zhang C Bollag G Shokat KM Rosen N RAF inhibitors transactivate RAF dimers and ERK signalling in cells with wild-type BRAF Nature 2010 464 427 30 10.1038/nature08902 20179705 PMC3178447

[B23] Heidorn SJ Milagre C Whittaker S Nourry A Niculescu-Duvas I Dhomen N Hussain J Reis-Filho JS Springer CJ Pritchard C Marais R Kinase-dead BRAF and oncogenic RAS cooperate to drive tumor progression through CRAF Cell 2010 140 209 21 10.1016/j.cell.2009.12.040 20141835 PMC2872605

[B24] Infante JR Fecher LA Nallapareddy S Gordon MS Flaherty KT Cox DS DeMarini DJ Morris SR Burris HA Messersmith WA Safety and efficacy results from the first-in-human study of the oral MEK 1/2 inhibitor GSK1120212 J Clin Oncol 2010 28 15 2503

[B25] Weeraratna AT RAF around the Edges - The Paradox of BRAF Inhibitors N Engl J Med 2012 366 271 273 10.1056/NEJMe1111636 22256810

[B26] Nazarian R Shi H Wang Q Kong X Koya RC Lee H Chen Z Lee MK Attar N Sazegar H Chodon T Nelson SF McArthur G Sosman JA Ribas A Lo RS Melanomas acquire resistance to B-RAF(V600E) inhibition by RTK or N-RAS upregulation Nature 2010 468 973 977 10.1038/nature09626 21107323 PMC3143360

[B27] Villanueva J Vultur A Lee JT Somasundaram R Fukunaga-Kalabis M Cipolla AK Wubbenhorst B Xu X Gimotty PA Kee D Santiago-Walker AE Letrero R D’Andrea K Pushparajan A Hayden JE Brown KD Laquerre S McArthur GA Sosman JA Nathanson KL Herlyn M Acquired resistance to BRAF inhibitors mediated by a RAF kinase switch in melanoma can be overcome by cotargeting MEK and IGF-1R/PI3K Cancer Cell 2010 18 683 695 10.1016/j.ccr.2010.11.023 21156289 PMC3026446

[B28] Orloff MJ Peskin GW Spontaneous rupture of the normal spleen: a surgical enigma Int Abstr Surg 1958 106 1 11 13495867

[B29] Sugahara K Togashi H Aoki M Mitsuhashi H Matsuo T Watanabe H Abe T Ohno S Saito K Saito T Shinzawa H Tanida H Ito M Takahashi T Spontaneous splenic rupture in a patient with large hepatocellular carcinoma Am J Gastroenterol 1999 94 276 278 10.1111/j.1572-0241.1999.00820.x 9934775

[B30] Smith WM Lucas JG Frankel WL Splenic rupture: a rare presentation of pancreatic carcinoma Arch Pathol Lab Med 2004 128 1146 1150 15387707 10.5858/2004-128-1146-SRARPO

[B31] Kyriacou A Arulai N Varia H Acute abdomen due to spontaneous splenic rupture as the first presentation of lung malignancy: a case report J Med Case Reports 2011 5 444 10.1186/1752-1947-5-444 PMC3178521 21899758

[B32] Karakousis CP Elias EG Spontaneous (pathologic) rupture of spleen in malignancies Surgery 1974 76 674 677 4213394

[B33] Richter RH Reingruber B Grüneis C Altendorf-Hofmann A Rupprecht H Spontaneous splenic rupture in metastatic malignant melanoma Chir 2001 126 630 631 10.1055/s-2001-16561 11519005

[B34] Buzbee TM Legha SS Spontaneous rupture of spleen in a patient with splenic metastasis of melanoma A case report. Tumori 1992 78 47 48 10.1177/030089169207800111 1609460

[B35] Sharma A Trivedi NR Zimmerman MA Tuveson DA Smith CD Robertson GB Mutant V599EB-Raf regulates growth and vascular development of malignant melanoma tumors Cancer Res 2005 65 2412 10.1158/0008-5472.CAN-04-2423 15781657

[B36] Bottos A Martini M Di Nicolantonio F Comunanza V Maione F Minassi A Appendino G Bussolino F Bardelli A Targeting oncogenic serine/threonine-protein kinase BRAF in cancer cells inhibits angiogenesis and abrogates hypoxia PNAS 2012 109 353 359 10.1073/pnas.1105026109 PMC3277561 22203991

[B37] Rak J Mitsuhashi Y Bayko L Filmus J Shirasawa S Sasazuki T Kerbel RS Mutant ras oncogenes upregulate VEGF/VPF expression: Implications for induction and inhibition of tumor angiogenesis Cancer Res 1995 55 4575 4580 7553632

